# Acute kidney injury in children with chronic kidney disease is associated with faster decline in kidney function

**DOI:** 10.1007/s00467-020-04777-z

**Published:** 2020-10-27

**Authors:** Nabil Melhem, Pernille Rasmussen, Triona Joyce, Joanna Clothier, Christopher J. D. Reid, Caroline Booth, Manish D. Sinha

**Affiliations:** 1 Department of Paediatric Nephrology, Evelina London Children’s Hospital, Guy’s & ST Thomas’ Foundation Hospitals NHS Trust, Westminster Bridge Road, London, SE1 7EH UK; 2 Kings College London, London, UK

**Keywords:** Acute kidney injury, Chronic kidney disease, Hypertension, Proteinuria, Progression, Risk factor, Children

## Abstract

**Background:**

This study aimed to investigate the association of acute kidney injury (AKI) with change in estimated glomerular filtration rate (eGFR) in children with advanced chronic kidney disease (CKD).

**Methods:**

Single centre, retrospective longitudinal study including all prevalent children aged 1–18 years with nondialysis CKD stages 3–5. Variables associated with CKD were analysed for their potential effect on annualised eGFR change (ΔGFR/year) following multiple regression analysis. Composite end-point including 25% reduction in eGFR or progression to kidney replacement therapy was evaluated.

**Results:**

Of 147 children, 116 had at least 1-year follow-up in a dedicated CKD clinic with mean age 7.3 ± 4.9 years with 91 (78.4%) and 77 (66.4%) with 2- and 3-year follow-up respectively. Mean eGFR at baseline was 29.8 ± 11.9 ml/min/1.73 m^2^ with 79 (68%) boys and 82 (71%) with congenital abnormalities of kidneys and urinary tract (CAKUT). Thirty-nine (33.6%) had at least one episode of AKI. Mean ΔGFR/year for all patients was − 1.08 ± 5.64 ml/min/1.73 m^2^ but reduced significantly from 2.03 ± 5.82 to − 3.99 ± 5.78 ml/min/1.73 m^2^ from youngest to oldest age tertiles (*P* < 0.001). There was a significant difference in primary kidney disease (PKD) (77% versus 59%, with CAKUT, *P* = 0.048) but no difference in AKI incidence (37% versus 31%, *P* = 0.85) between age tertiles. Multiple regression analysis identified age (*β* = − 0.53, *P* < 0.001) and AKI (*β* = − 3.2, *P* = 0.001) as independent predictors of ΔGFR/year. 48.7% versus 22.1% with and without AKI reached composite end-point (*P* = 0.01).

**Conclusions:**

We report AKI in established CKD as a predictor of accelerated kidney disease progression and highlight this as an additional modifiable risk factor to reduce progression of kidney dysfunction.

Graphical abstract
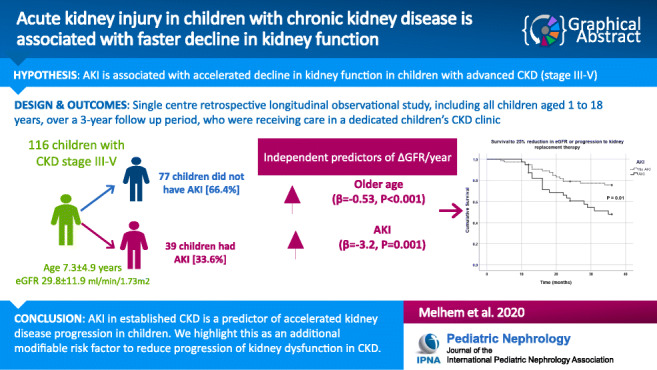

**Electronic supplementary material:**

The online version of this article (10.1007/s00467-020-04777-z) contains supplementary material, which is available to authorized users.

## Introduction

There has been extensive interest in understanding and controlling modifiable risk factors for kidney disease progression in chronic kidney disease (CKD) in both adults and children. Mortality in children with kidney failure with replacement therapy is up to 30 times higher than the general population, making our understanding and management of pre-dialysis CKD crucial to achieving improved outcomes [1]. Hypertension and proteinuria are established modifiable predictors of kidney disease progression in both adults and children with CKD [2–5]. Additional risk factors that have been associated with kidney disease progression in children include anaemia, hypoalbuminemia, hyperphosphatemia, and vitamin D deficiency [6–8]. Clinical strategies for preserving nephrons in children with CKD have primarily focused on ameliorating these risk factors.

There remains a paucity of data evaluating the effects of acute kidney injury (AKI) in children with CKD, and its possible role in accelerating kidney disease progression. This is despite an increasing understanding that AKI and CKD are not distinct clinical entities but closely interconnected [9]. One can lead to the other and both these syndromes share risk factors for cardiovascular disease and increased patient mortality and morbidity [10].

Our objective in this study was to evaluate the impact of AKI on disease progression in children with non-dialysis stages 3–5 of CKD.

## Methods

### Study population

We report a single-centre retrospective longitudinal observational study, including all children aged 1 year to 18 years, over a 3-year period of follow-up (April 2016 to March 2019) who were receiving care in a dedicated children’s CKD clinic. The CKD clinic at the Evelina London Children’s Hospital, London, has a team of three paediatric nephrologists (CJB, NM, JC), a CKD senior nurse specialist (PR), and a paediatric renal dietician (TJ), with additional input as required from the wider nephro-urology department.

### Inclusion and exclusion criteria

Patients were included if all of the following criteria were met: (i) under the care of the specialist CKD clinic for a minimum of 1 year, (ii) eGFR < 60 ml/min/1.73 m^2^ at baseline, and (iii) minimum of 3 creatinine measurements per year were available; all performed as part of routine clinical care. Patients were excluded from the study if (i) CKD was being managed on a conservative care pathway or (ii) follow-up in the dedicated CKD clinic was for < 1 year.

### Data collection

All included patients routinely attended the specialist CKD clinic at a minimum frequency of 4 months. Pre-defined clinical and laboratory parameters were collated from the electronic hospital records for each patient at annual intervals over a recent 3-year period. For all patients we aimed to collect data including (i) haemoglobin, serum creatinine, albumin, urea, corrected calcium, phosphate, intact parathyroid hormone (PTH), and urine protein creatinine ratio (uPCR) on a random urine specimen. The estimated glomerular filtration rate (eGFR) in ml/min/1.73 m^2^ was calculated using the modified Schwartz formula with our previously described correction factor [11–13], and CKD stage defined according to published definitions [14]. (ii) All office blood pressure measurements were performed manually by trained nursing and medical staff during routine clinical care using an aneroid sphygmomanometer. Blood pressure *z*-score was calculated using data from the fourth report on the diagnosis, evaluation, and treatment of high blood pressure in children and adolescents [15]. Data regarding antihypertensive medication, including if patients were on renin angiotensin aldosterone inhibitors, was collected. Ethnicity was defined as reported by the patient/family and recorded on the electronic patient record.

### Acute kidney injury

The incidence, cause, severity, and outcome of AKI were recorded from the electronic health record. To differentiate AKI from rapid disease progression, an increase in creatinine level was required to show reversibility. Reversal to baseline was defined as a return of creatinine value to within < 10% of previous baseline serum creatinine measurement. An episode of AKI was defined as per KDIGO criteria [16]. A creatinine rise of 1.5–1.9 times baseline was classified as stage 1 of AKI, a rise in creatinine of 2–2.9 times baseline was classified as stage 2 of AKI, and a rise in creatinine of > 3 times baseline or an increase in creatinine to 354 μmol/L (4 mg/dl) was classified as stage 3 of AKI. The stage 3 definition of a reduction in eGFR to < 35 ml/min/1.73 m^2^ was omitted from our criteria to avoid bias in our population with already very low GFR. Additionally, we also classified as AKI if there was a rise in serum creatinine of > 20% from baseline, as we considered this a clinically significant increase in this low GFR population. Episodes of AKI were identified from health records and were analysed for change in serum creatinine level in each patient when compared with previous measurements.

### Outcome measures

The following outcome measures were evaluated: (i) the annualised change in eGFR (ΔGFR/year), calculated by dividing the overall change in eGFR from baseline to last follow-up by the number of years observed during the study period, and (ii) the composite end-point including 25% reduction in eGFR from baseline or progression to kidney replacement therapy. Annual GFR values were distinct from change in creatinine (and GFR) at the time of any AKI episodes as they did not show evidence of reversal towards a previous baseline and were taken whilst patients were clinically well.

### Statistical analysis

Subject characteristics are summarised as means ± standard deviation (SD) or median and interquartile range (IQR) as appropriate. We divided our population in to three equally distributed age tertiles for comparison of disease progression between different developmental stages. Multiple regression analysis was used to examine the association of potential independent predictors with ΔGFR/year. Variables were considered if clinically relevant or known to be associated with disease progression. The final regression model included age, sex, ethnicity, primary kidney disease (PKD) as CAKUT/glomerular/other, stage of CKD at baseline, AKI episode (yes/no), angiotensin converting enzyme (ACE) inhibitor use, and systolic blood pressure *z*-score. Goodness of fit was expressed as the adjusted *r*^2^, and multicollinearity was evaluated using the variance inflation factor.

The proportion of subjects to reach composite end-point was analysed using Kaplan–Meier survival analysis with log-rank test to examine the differences in the rates of end points between those with AKI and those without AKI. Cox proportional-hazard analysis was performed to assess the impact of other potential variables on reaching composite end-point. Variables included in the Cox proportional-hazard model were AKI occurrence (Yes/No), sex, ethnicity (white/other), PKD (CAKUT/other), low baseline GFR (< 30 ml/min/1.73m^2^/> 30 ml/min/1.73 m^2^), RAAS inhibition (Yes/No), BP category (optimal control; < 50th centile/suboptimal control; > 50th centile), and age. The level of statistical significance selected was *P* < 0.05 throughout. Statistical analyses were performed using IBM SPSS statistics version 24.

## Results

A total of 147 patients with stages 3–5 of CKD attended the dedicated specialist CKD clinic over the 3-year study period, 116 (79%) of whom were included in this study. Follow-up was available for 116 (100%), 91 (78.4%), and 77 (66.4%) patients at 1, 2, and 3 years. We excluded 31 (21.1%) patients from analysis, including (i) six who were managed on a conservative care pathway, (ii) six with an eGFR > 60 ml/min/1.73m^2^, (iii) 17 who had less than one year follow-up, and (iv) two with insufficient creatinine measurements performed. Characteristics of included versus excluded patients are displayed in Table 1. Excluded patients had higher eGFR and were younger at last follow-up and were excluded as they had GFR > 60 ml/min/1.73 m^2^ and/or follow-up of less than 12 months.

**Table 1:** Demographics and clinical characteristics of children included and excluded in the study.

	Included patients (*n* = 116)	Excluded patients (*n* = 31)		*P*
Age at last follow up (years)*	9.74 ± 4.86	5.57 ± 5.29		< 0.001
eGFR at last follow-up (ml/min/1.73 m^2^)*	28.1 ± 14.3	42.1 ± 32.3		0.02
Gender (M/F)	79/39	20/11		0.71
Ethnicity (white/black/Asian/other, %)	58/15/15/12	51/10/39/0		0.01
PKD (CAKUT/glomerular/other, %)	71/3/26	55/10/35		0.16

*Data shown as mean ± SD

*eGFR* estimated glomerular filtration rate, *PKD* primary kidney disease, *CAKUT* congenital abnormalities of kidneys and urinary tract

### All patients at baseline and at last follow-up

Of the 116 included patients, mean ± SD age at baseline was 7.3 ± 4.9 years and mean ± SD eGFR was 29.8 ± 11.9 ml/min/1.73 m^2^, of whom 79 (68.1%) were boys and 82 (70.6%) had CAKUT. Fifty-five (47.4%), 48 (41.4%), and 13 (11.2%) patients had stages 3, 4, and 5 of CKD. Mean ± SD systolic and diastolic BP *z*-score was − 0.44 ± 1.43 and − 0.41 ± 1.60 respectively. A total of 33 (28.4%) patients had random uPCR measurements with a median (IQR) level of 96 (50, 257) mg/mmol.

At last follow-up, mean ± SD age was 9.7 ± 4.9 years and mean ± SD eGFR was 28.1 ± 14.3 ml/min/1.73 m^2^. Children with CAKUT had a mean ± SD ΔGFR/year of − 0.22 ± 4.35 ml/min/1.73 m^2^ (95% CI − 1.18 to 0.74), whereas those with other causes of PKD had a faster decline of − 3.17 ± 7.62 ml/min/1.73 m^2^ (95% CI − 5.83 to − 0.51), *P* = 0.04. A total of 40/116 patients (34.5%) showed improvement in eGFR at last follow-up when compared with baseline, and the median (IQR) age of this group was 6.5 years (2.9, 12.0). Fifty-five (47.4%) patients had random uPCR measurements with a median (IQR) level of 100 (41, 204) mg/mmol. Mean ± SD systolic and diastolic BP *z*-score was 0.17 ± 1.25 and 0.27 ± 1.15 respectively. Thirty-three (28.4%) were on RAAS inhibitor therapy in combination with other antihypertensive medication, and 20 (17.2%) were on RAAS inhibitor therapy alone. Ten (18%) patients with stage 3 CKD, 7 (21%) patients with stage 4 CKD, and 3 (10%) patients with stage 5 CKD continued on RAAS inhibition at last follow-up.

### Results by age tertiles

**Fig. 1:**
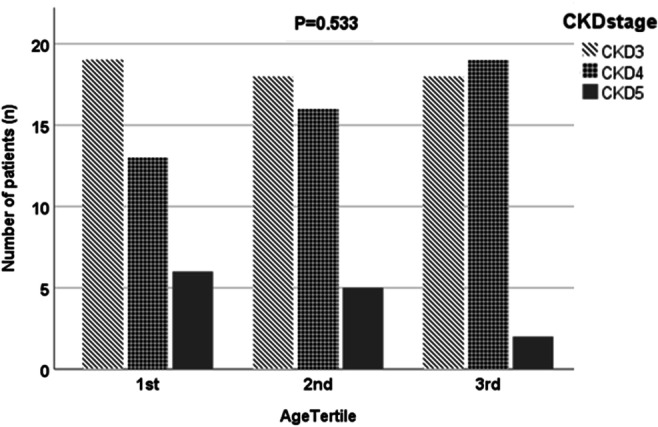
Baseline CKD stage distribution in each age tertile with tertile 1 including the youngest children. CKD chronic kidney disease

Demographic and clinical characteristics divided into age tertiles are displayed in Table 2 with stages of CKD by age tertiles shown in Fig. 1. The mean ± SD ∆GFR/year for tertiles 1, 2, and 3 was 2.03 ± 5.82 ml/min/1.73 m^2^, − 1.21 ± 3.39 ml/min/1.73 m^2^, and − 3.99 ± 5.78 ml/min/1.73 m^2^ respectively (*P* < 0.001). There was significant difference in PKD by age tertiles with 76%, 77%, and 59% patients with CAKUT in tertiles 1, 2, and 3 respectively (*P* = 0.048) and significant difference in baseline systolic BP *z*-scores by age tertiles with *z*-scores of 0.48 ± 1.43, − 0.39 ± 0.88, and − 0.92 ± 1.76 in tertiles 1, 2, and 3 respectively (*P* = 0.02). There were no significant differences between tertiles for sex, ethnicity, height *z*-score, episodes of AKI, ACE inhibitor use, haemoglobin, albumin, intact PTH, or vitamin D concentrations (Table 2).

**Table 2:** Demographics and clinical characteristics of children included divided by age tertile at baseline. All data shown as mean ± SD unless specified

	All (*n* = 116)	1st tertile (*n* = 38)	2nd tertile (*n* = 39)	3rd tertile (*n* = 39)				*P*
Age range at follow-up (years)	1.3–17.0	1.3–6.8	6.9–12.6	12.7–17.0				
Age at baseline (years)	7.31 ± 4.91	1.51 ± 1.40	7.41 ± 1.80	12.86 ± 1.70				
Age at last follow up (years)	9.7 ± 4.9	3.9 ± 1.7	10.0 ± 1.6	15.2 ± 1.3				
Time since diagnosis of CKD (years)	8.59 ± 11.29	3.76 ± 1.54	11.39 ± 18.00	10.49 ± 4.95				0.005
Sex (M/F)	79/37	24/14	28/11	27/12				0.71
Ethnicity (white/black/asian/other, %)	68/17/17/14	50/21/11/18	64/15/10/10	62/8/23/8				0.24
PKD (CAKUT/glomerular/other, %)	71/3/26	70/0/24	77/0/23	59/10/31				0.048
Height (*z*-score)	− 0.90 ± 1.19	− 0.94 ± 1.21	− 1.14 ± 1.35	− 0.62 ± 0.93				0.14
SBP (*z*-score)	− 0.44 ± 1.43	0.49 ± 1.09	− 0.39 ± 0.88	− 0.92 ± 1.76				0.02
DBP (*z*-score)	− 0.41 ± 1.60	0.15 ± 1.04	− 0.54 ± 1.77	− 0.54 ± 1.62				0.28
CKD stage (3/4/5, %)	47/41/11	50/34/16	46/41/13	46/49/5				0.53
ACEi, *n* (%)	20 (17.2)	3 (7.9)	7 (17.9)	10 (25.6)				0.12
ACEi + other antihypertensive, *n* (%)	33 (28.4)	9 (23.7)	6 (15.4)	18 (46.2)				0.01
Vitamin D preparations, *n* (%)	68 (58.6)	13 (34.2)	27 (69.2)	28 (58.6)				0.001
Trimethoprim, *n* (%)	16 (13.8)	5 (13.2)	7 (17.9)	4 (10.3)				0.61
Anticholinergic/antimuscarinic, *n* (%)	24 (20.7)	8 (21.2)	8 (20.5)	8 (20.5)				0.99
Phosphate binding agent, *n* (%)	52 (44.8)	8 (21.1)	22 (56.4)	22 (56.4)				0.002
Sodium bicarbonate, *n* (%)	80 (69)	27 (71.1)	28 (71.8)	25 (64.1)				0.72
AKI*, *n* (%)	39 (33.6)	14 (36.8)	12 (30.8)	13 (33.3)				0.85
Haemoglobin (g/l)	120.3 ± 15.5	116.4 ± 19.3	121.3 ± 10.5	123.2 ± 15.1				0.14
Albumin (g/l)	43.9 ± 3.9	43.7 ± 3.5	44.2 ± 2.9	43.6 ± 5.1				0.77
Adjusted calcium (mmol/l)	2.47 ± 0.13	2.56 ± 0.12	2.44 ± 0.12	2.41 ± 0.08				< 0.001
Phosphate (mmol/l)	1.41 ± 0.29	1.61 ± 0.30	1.34 ± 0.19	1.29 ± 0.28				< 0.001
iPTH (ng/l)	97.6 ± 102.8	80.8 ± 50.9	90.3 ± 87.4	120.8 ± 144.0				0.21
25-hydroxy vitamin D (nmol/l)	72.4 ± 31.1	79.2 ± 29.5	77.8 ± 37.4	72.4 ± 31.1				0.07
Proportion improved GFR (%)	34.5	57.9	23.1	23.1				0.001
Annualised ΔGFR* (ml/min/1.73m^2^/year)	− 1.08 ± 5.64	2.03 ± 5.82	− 1.21 ± 3.39	− 3.99 ± 5.78				< 0.001

*CKD* chronic kidney disease, *PKD* primary kidney disease, *CAKUT* congenital abnormalities of kidneys and urinary tract, *SBP* systolic blood pressure, *DBP* diastolic blood pressure, *ACEi* angiotensin converting enzyme inhibitor, *AKI* acute kidney injury, *iPTH* intact parathyroid hormone, *GFR* glomerular filtration rate

*Over the study duration of 3 years

### Acute kidney injury

In total there were 56 identified episodes of AKI amongst 39 (33.6%) patients. During the observation period, 26 (22.4%) patients had a single AKI episode, 9 (7.8%) patients had 2 discrete episodes, and 4 (3.4%) patients had 3 discrete episodes. All episodes showed reversal of creatinine levels towards baseline. Exactly 47 out of 56 (84%) episodes of AKI showed reversal of creatinine levels back to within 10% of baseline value. Causes of AKI included urinary tract infections (26.8%); dehydration associated with vomiting, diarrhoea, and upper respiratory tract infections (26.8%); elective surgery (14.3%); obstructive uropathy not related to infection (5.4%); kidney toxicity secondary to medication (1.8%); and reversible primary disease decompensation or unidentified cause (19.6%). Of the 56 episodes of AKI, 29 (51.8%), 5 (8.9%), and 16 (28.6%) were stages 1, 2, and 3 of AKI, and 6 (10.7%) had an AKI episode which did not meet AKIN criteria that was > 20% but < 50% rise of creatinine from baseline.

**Fig. 2:**
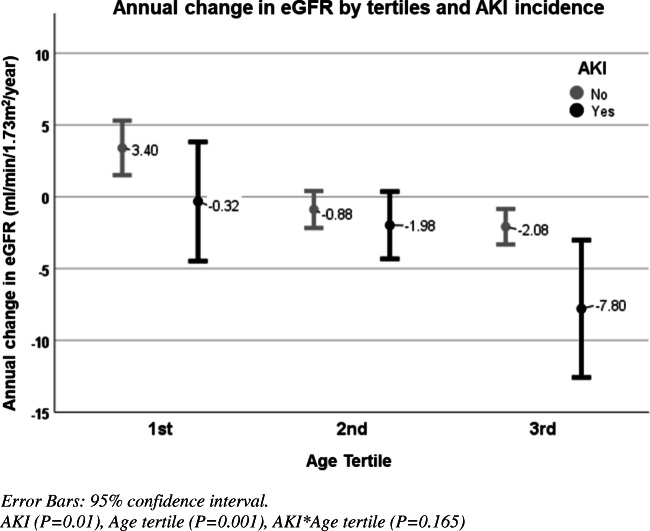
Changes in annual eGFR (ml/min/1.73 m^2^) between different age tertiles in those with AKI and those with no AKI episodes. A two-way ANOVA was conducted to examine the effect of age tertile and acute kidney injury incidence on ∆GFR/year. eGFR estimated glomerular filtration rate, AKI acute kidney injury

The mean ∆GFR/year in the group with no AKI was 0.05 ± 4.27 ml/min/1.73 m^2^ (95% CI − 0.92 to 1.02) compared with − 3.32 ± 7.22 ml/min/1.73 m^2^ (95% CI − 5.66 to − 0.98) in the group with AKI (*P* = 0.01) (Fig. 2). ∆GFR/year for those exposed to stage 1 of AKI and stage 2/3 of AKI was − 2.37 ± 6.26 ml/min/1.73 m^2^ (95% CI − 5.08 to 0.34) and − 4.69 ± 8.45 ml/min/1.73m^2^ (95% CI − 9.19 to − 0.19) respectively. When comparing the mean ∆GFRs/year in those with no AKI, stage 1 of AKI, and stage 2 or 3 of AKI, severity was associated with a significantly higher rate of decline (*P* = 0.004). In those with multiple episodes of AKI, there was no significant difference in ∆GFR/year when compared with those with a single episode of AKI (*P* = 0.11). There was no significant difference found in sex, ethnicity, age group, PKD, ethnicity, baseline CKD stage, level of BP, ACE inhibitor use, or evaluated laboratory indices between the two groups (Table 3).

**Table 3:** Comparison of characteristics of subjects with AKI versus subjects with no AKI

	AKI (*n* = 39)	No AKI (*n* = 77)				*P*
Age (mean ± SD, range)	9.4 ± 5.0	9.9 ± 4.8				0.62
Sex (M/F)	26/13	53/24				0.81
Ethnicity (white/black/Asian/other, %)	56/10/18/15	60/17/13/10				0.62
PKD (CAKUT/glomerular/other, %)	74/8/18	69/1/30				0.1
SBP (*z*-score)	0.34 ± 0.72	0.08 ± 1.45				0.20
DBP (*z*-score)	0.27 ± 1.31	0.28 ± 1.07				0.96
Baseline CKD stage (3/4/5, %)	41/49/10	51/38/12				0.52
ACEi (%)	10	21				0.16
Haemoglobin (g/l)	112.4 ± 14.4	124.3 ± 14.5				0.95
Albumin (g/l)	43.2 ± 3.9	44.2 ± 3.9				0.34
Adjusted calcium (mmol/l)	2.48 ± 0.13	2.47 ± 0.13				0.62
Phosphate (mmol/l)	1.49 ± 0.30	1.37 ± 0.29				0.58
iPTH (ng/l)	91.7 ± 56.8	100.6 ±120.0				0.52
25-hydroxy vitamin D (nmol/l)	76.0 ± 28.0	70.0 ± 33.1				0.56
Proportion improved GFR (%)	23.1	40.3				0.07
Annualised ΔGFR (ml/min/1.73m^2^/year)	-3.32 ± 7.22	0.05 ± 4.27				0.01

*AKI* acute kidney injury, *PKD* primary kidney disease, *CAKUT* congenital abnormalities of kidneys and urinary tract, *SBP* systolic blood pressure, *DBP* diastolic blood pressure, *CKD* chronic kidney disease, *ACEi* angiotensin converting enzyme inhibitor, *iPTH* intact parathyroid hormone, *GFR* glomerular filtration rate

In multivariable regression analyses, age (*β* = − 0.53, *P* < 0.001) and presence of AKI (*β* = −3.2, *P* < 0.001) were independent predictors of ΔGFR/year (adjusted *R*^2^ 0.28, *P* < 0.001; variance inflation factor was 1.1) (Table 4).

**Table 4:** Multivariable regression analyses to predict ΔGFR/year from relevant independent variables. Model adjusted *R*^2^ = 0.28

Variable	Coefficient (*β*)	SEM	95% confidence interval for *β*	*P*
Age (years)	− 0.53	0.10	− 0.72 to − 0.34	< 0.001
Sex (M/F)	0.34	0.94	− 1.52–2.20	0.72
Ethnicity (white/black/Asian/other)	− 0.43	0.40	− 1.22–0.36	0.28
Aetiology (CAKUT/glomerular/other)	− 0.80	0.52	− 1.83–0.23	0.13
Baseline CKD stage (3/4/5)	0.63	0.65	− 0.66–1.92	0.34
Systolic BP (*z*-score)	− 0.05	0.35	− 0.75–0.65	0.88
ACEi/ARB (yes/no)	− 2.15	1.18	− 4.50–0.20	0.07
AKI (yes/no)	− 3.20	0.92	− 0.50 to −1.37	0.001

*GFR* glomerular filtration rate, *CAKUT* congenital abnormalities of kidneys and urinary tract, *CKD* chronic kidney disease, *BP* blood pressure, *ACEi* angiotensin converting enzyme inhibitor, *ARB* angiotensin receptor blocker, *AKI* acute kidney injury

**Fig. 3:**
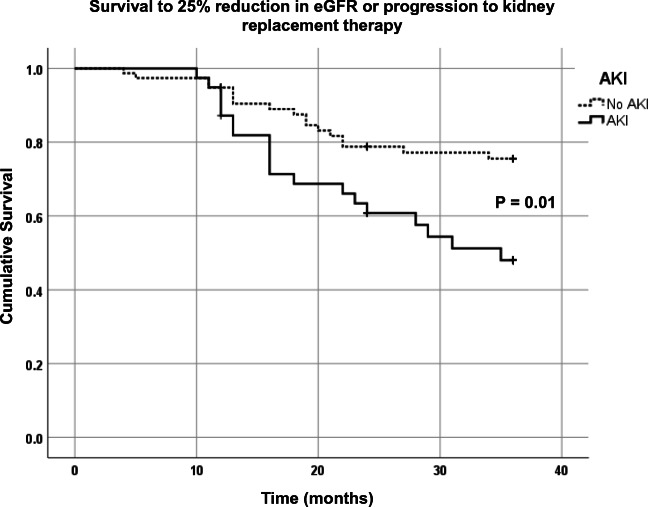
Kaplan–Meier plot showing time to composite end-point of 25% reduction in GFR or progression to kidney replacement therapy in those with AKI versus those with no AKI. eGFR estimated glomerular filtration rate, AKI acute kidney injury

During the observation period, 19 of 39 patients (48.7%) versus 17 of 77 patients (22.1%) with and without AKI reached composite end-point (*P* = 0.01 log-rank test) (Fig. 3). The hazard ratio for progression to composite end-point following AKI was 2.78 (95% confidence interval (CI) 1.37 to 5.62, *P* = 0.004). Other variables associated with an increased risk of reaching the composite end-point included low baseline GFR (hazard ratio 2.23, 95% CI 1.07 to 4.66, *P* = 0.033) and older age (hazard ratio 1.11, 95% CI 1.03 to 1.21, *P* = 0.01).

## Discussion

The main finding of our study is that in children with CKD, episodes of AKI are associated with accelerated kidney disease progression. Nearly twice as many patients with AKI were observed to have progressive kidney dysfunction and reach the composite study endpoint when compared with those who had no AKI. These findings are particularly relevant as traditional targets to reduce rate of progression of CKD, such as level of BP and ACE inhibitor use, were similar across patient groups.

Our findings are in keeping with previous data in adults demonstrating a link between AKI and CKD where several observational studies demonstrate that AKI, even when mild, can predispose to subsequent CKD [9, 17–20], with the risk of kidney failure ~ 13 times higher than those without AKI [19]. Similarly in the paediatric population, CKD can develop in children who had normal kidney function before AKI, although this is often in the context of cardiac surgery or AKI requiring temporary dialysis [21, 22]. One study of children admitted to intensive care showed that exposure to AKI increased the risk of CKD at 5-year follow-up, with a hazard ratio of 2.2 for stage 1 of AKI and 2.5 for stages 2–3 of AKI [23]. Similarly, we also observed that increasing severity of AKI was associated with accelerated disease progression. Another study showed that recurrent UTIs in children with CKD associates with kidney disease progression, although AKI in this study was not specifically implicated [24]. In our population, we observed that having repeated episodes of AKI was not associated with faster decline in kidney function than a single episode. This is in contrast to previous reports in adults that highlight cumulative AKI exposure may hasten CKD progression [10, 25]. These differences may have been due to the limited numbers of patients with multiple AKI episodes for analysis in our cohort. It is interesting to note that whilst there was no statistical difference in PKD between those with AKI and those with no AKI, 30% with AKI versus 18% with no AKI were classified as “other” PKD. These findings may reflect the issues around categorisation of PKD in our study but may additionally be suggestive of differences in susceptibility to AKI of different kidney conditions and need further exploration in future prospective work.

AKI and CKD are not distinct clinical entities but closely interconnected [9]. As well as AKI predisposing to CKD progression, CKD also increases the risk of AKI exposure which leads to faster progression to kidney failure. In one adult study of hospitalised patients requiring dialysis for management of AKI compared with those who did not, worse GFR and presence of proteinuria were identified as risk factors [26]. Further, whilst we did not compare our CKD cohort to children with normal kidney function, 33.6% of our population developed AKI. This is notably high compared with large UK population cohorts which show a general population-based AKI incidence of approximately 1.4% in a given year [27]. This suggests that children with CKD are also at higher risk of AKI exposure.

Our study population is comparable with the ESCAPE and CKiD study cohorts, with regard to rates of eGFR decline [2, 23, 28]. In our study we have shown results for achieved BP control using clinic BP measurements only as this was uniformly measured across all age groups. Using mean clinic BP measurements we observed this was well controlled in our population and maintained below the 50th percentile at baseline and below the 60th percentile at last follow up. Twenty-four-hour ambulatory BP monitoring at these levels of clinic BP measurements is less likely to have resulted in changes in BP management as shown in a recent study by the CKiD investigators [29]. Additionally, our study cohort had an eGFR of 28 ml/min/1.73 m^2^ at follow-up and escalation of RAAS inhibitor therapy would be similarly less likely. Unfortunately, proteinuria was not measured systematically in the majority of patients in this study at baseline and ~ 55% at follow up. This likely reflects the difficulty in collecting urine specimens in younger patients, but also the markedly lower GFR in our study cohort where RAAS inhibitor therapy escalation based on the degree of proteinuria may not be clinically appropriate, or when qualitative urinalysis testing in clinic did not show proteinuria. The rate of kidney disease progression has previously been shown to be higher in patients with lower baseline GFR [8]. Although we do not report this, the relatively lower number of patients with glomerular disease and the younger population may be possible causes for this.

In addition to AKI, we observed a significant association between age and rate of GFR decline in CKD. This is in part explained by the longer disease duration in the oldest patients and an increased prevalence of progressive glomerular disease. Conversely the majority of patients in the youngest age group showed improving kidney function which is in keeping with a similar cohort of 176 children with CKD where 82% showed early improvement in kidney function until a median age of 3.2 years old [24]. Nephrogenesis ceases at 32-week gestation, and therefore, infants with CAKUT have a lower nephron endowment before birth, correlating to poorer kidney prognosis. Nonetheless, kidney function continues to increase after birth due to haemodynamic compensatory mechanisms and nephron hypertrophy [31–33]. Our findings highlight that these formative years are an important developmental period for nephrons in this patient group, which may determine long-term kidney function. In our youngest group, whilst those with no AKI showed substantial improvement in kidney function, the AKI group showed a contrasting decline. This suggests that vital compensatory mechanisms recruited during infancy in low nephron number states may be detrimentally arrested by AKI. This observation needs further exploration in future work but demonstrates that the formative years of improving kidney function are both vital and fragile, and AKI in this time-period may have long-term ramifications.

The aetiology of AKI in children with CKD is not well documented and is often unclear. One of the strengths of this study is that we have been able to identify cause of AKI from clear documents in the patient records. Drug-related kidney toxicity is a recognised leading cause of AKI in hospitalised children [34, 35]. In contrast, we report very low (~ 2%) AKI related to drug-induced kidney toxicity. This may reflect our standard advice to temporarily stop ACEi/ARB and to avoid NSAIDs during an episode of acute illness that could lead to hypovolaemia such as gastroenteritis or suspected sepsis. We also believe outcomes are improved by ready access of families to expert advice, whenever needed, from our Clinical Nurse Specialist and Consultant team. As we did not collect any formal systematic information regarding this we are unable to comment any further.

The reno-protective effect of RAAS blockade in adult patients has been well established in several randomised controlled trials, independently of BP control with other antihypertensive medications [2, 36]. This study shows for the first time that potentially avoidable AKI is a further modifiable risk factor for kidney disease progression in children with CKD. Whilst prospective multi-centre data are needed to build upon these findings, we recommend clinical strategies to mitigate against the common causes of AKI highlighted in our study. This also leads to the question of long-term RAAS inhibitor use in CKD. There is potential for causing longer-term harm by their use in CKD patients who experience AKI, or conversely by discontinuing this therapy prematurely due to perceptions of damage caused by their use []. We found no association between the number or severity of AKI episodes with the use of RAAS inhibition. Whilst many physicians advise cessation of ACE/ARB inhibition during AKI, the evidence is lacking for this practice and the point at which the deleterious effects of RAAS inhibition outweigh protective actions against tubular injury and inflammation remains a matter of conjecture [37]. This relationship needs further evaluation, especially in the context of AKI in CKD.

An area of research to better understand the relationship between AKI and CKD is the role of novel biomarkers for predicting CKD progression. Emerging candidate biomarkers for CKD progression include surrogates of tubulointersitital injury (NGAL, IL-18, KIM-1, EGF), tubulointerstitial fibrosis (MMP-9, PIIINP, TGF-β1, BMP-7), and inflammation (MCP-1, TNFR 1/2, suPAR) [38]. In paediatrics, studies have been limited to small cross-sectional analyses and biomarkers which accurately predict CKD progression are still sought after. Progress in this field has the potential to better understand the relationship between the interconnected entities of AKI and CKD, to guide and predict response to therapies, and to predict long-term outcomes.

There are important limitations relating to the retrospective nature of this study, including the following: (i) There were limited numbers of patients with glomerular disease. Patients with glomerular disease have a more rapid progression of kidney disease compared with patients with CAKUT. Some patients with rapid disease progression may not have achieved the 1-year follow-up in the chronic kidney disease clinic required for inclusion. Additionally, AKI may be more relevant to patients at earlier stages of CKD as their potential to recover kidney function following AKI may be blunted due to ongoing glomerular inflammation at later stages. This is an important subgroup of patients to investigate, and more work is needed to prospectively examine this cohort. Notably, the aetiology of CKD did not associate with rate of eGFR decline in our study, but this may have been due to the small numbers of glomerular disease patients included. (ii) Whilst most episodes of AKI were captured on the electronic patient record, some will have been missed from patients not seeking medical attention for mild AKI, or presenting to other hospitals for acute management. We believe very few of these episodes would not have been reported as it is our standard practice to update our hospital records regarding admissions to other hospitals, and the clinical management is usually discussed by telephone and guided by our team. We accept though that there may have been variation to this practice resulting in an under-estimation of AKI in this population. (iii) The retrospective nature of our study means that unfortunately we cannot be certain that all episodes of UTI/diarrhoea and vomiting were uniformly recorded. Hence, such clinical episodes which did not result in AKI were not analysed due to incomplete recording of this data outside of our organisation. We recognise that knowledge of these episodes may inform development of a more pro-active management plan including fluid management and stopping ACE inhibitors that might mitigate risks of AKI-induced GFR decline. This should be the focus of further prospective work. (iv) Tanner staging of puberty was not routinely performed in our study cohort and when performed not formally documented. We accept that faster decline in GFR in the oldest patients may be additionally related to pubertal development. (v) We chose the composite endpoint of 25% reduction in GFR or commencement of kidney replacement therapy similar to the ESCAPE trial, but accept that this may not always be clinically meaningful, especially in those with better GFR. Evaluation of single or a panel of biomarkers may be a useful adjunct to evaluate relevant outcomes following AKI in this population. (vi) Lastly, the follow-up period of 3 years limits extrapolation to much longer-term outcomes, especially in the youngest age group.

## Conclusion

In conclusion, the most significant finding of this study is that AKI in patients with CKD is significantly associated with an increase in the rate of kidney disease progression. Drug-induced kidney toxicity as a cause of AKI is less prevalent in this population. In addition to strict blood pressure control and minimisation of proteinuria, strategies to prevent AKI in this vulnerable population are vital to preserve longer-term kidney function. Further prospective studies are needed in this important area to build upon these findings.

## Electronic supplementary material

ESM 1 (PPTX 121 kb)
