# Supplementary material for: Acute kidney injury in children with chronic kidney disease is associated with faster decline in kidney function
Source: Pediatr Nephrol. 2020 Oct 27;36(5):1279–88. doi: 10.1007/s00467-020-04777-z (PMC8009790; doi:10.1007/s00467-020-04777-z)
Supplement: Supplementary file 1 — (PPTX 121 kb) [file 467_2020_4777_MOESM1_ESM.pptx]

## Slide 1
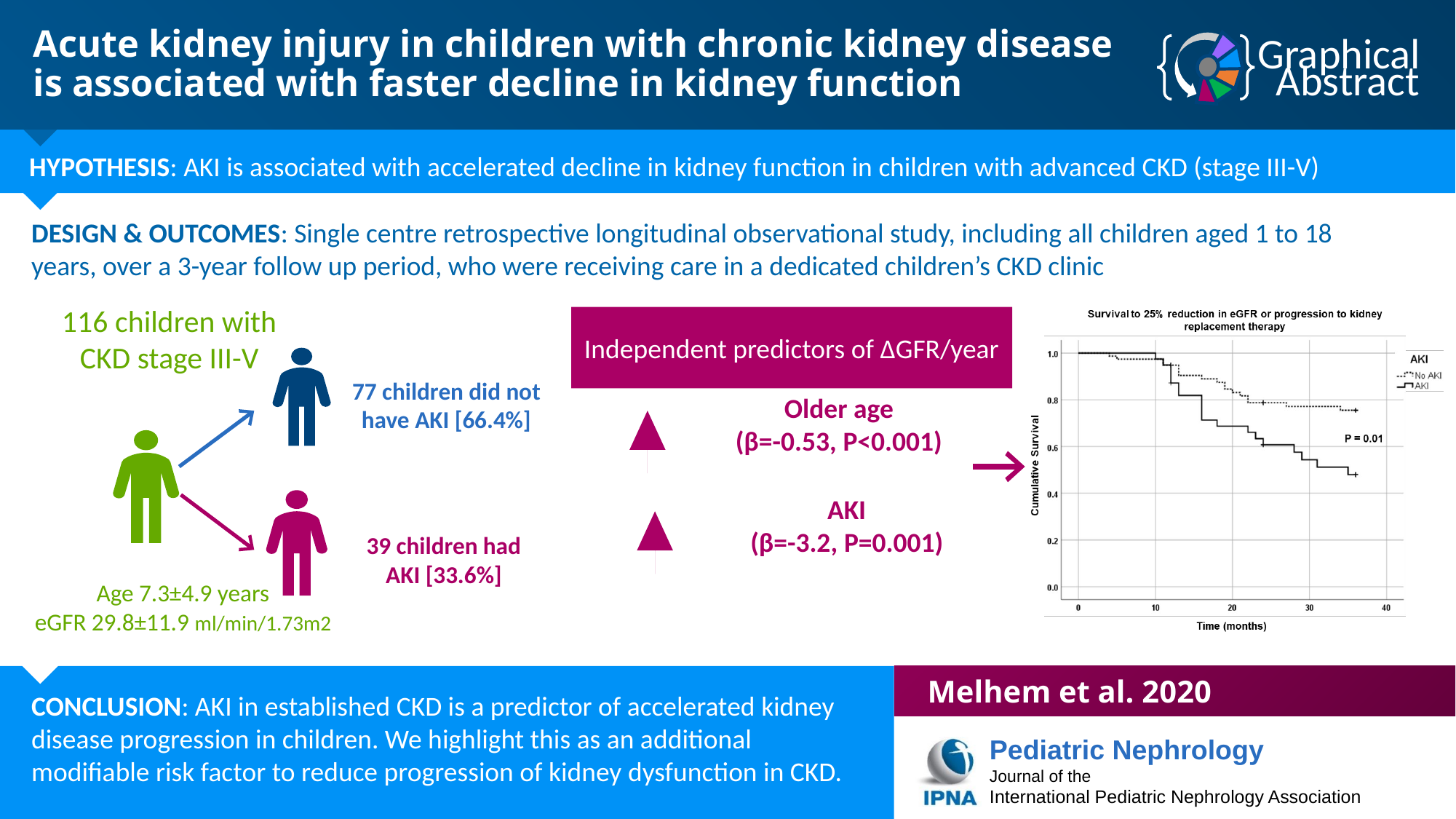

Acute kidney injury in children with chronic kidney disease is associated with faster decline in kidney function
HYPOTHESIS: AKI is associated with accelerated decline in kidney function in children with advanced CKD (stage III-V)
DESIGN & OUTCOMES: Single centre retrospective longitudinal observational study, including all children aged 1 to 18 years, over a 3-year follow up period, who were receiving care in a dedicated children’s CKD clinic
116 children with CKD stage III-V
Independent predictors of ΔGFR/year
77 children did not have AKI [66.4%]
Older age
(β=-0.53, P<0.001)
AKI
(β=-3.2, P=0.001)
39 children had AKI [33.6%]
Age 7.3±4.9 years
eGFR 29.8±11.9 ml/min/1.73m2
Melhem et al. 2020
CONCLUSION: AKI in established CKD is a predictor of accelerated kidney disease progression in children. We highlight this as an additional modifiable risk factor to reduce progression of kidney dysfunction in CKD.
